# Photodynamic Therapy for Colorectal Cancer: A Systematic Review of Clinical Research

**DOI:** 10.1177/15533506221083545

**Published:** 2022-04-15

**Authors:** Keegan Guidolin, Lili Ding, Han Yan, Marina Englesakis HBA, Sami Chadi, Fayez Quereshy, Gang Zheng

**Affiliations:** 1 Department of Surgery, 7938 University of Toronto, Toronto, ON, Canada; 2 Institute of Biomedical Engineering, 7938 University of Toronto, Toronto, ON, Canada; 3 10051 Princess Margaret Cancer Centre, Toronto, ON, Canada; 4 7989 University Health Network, Toronto, ON, Canada

**Keywords:** photodynamic therapy, colon cancer, rectal cancer, colorectal cancer, neoadjuvant therapy, adjuvant therapy, photosensitizer, photofrin, phototherapy

## Abstract

**Background:**

Photodynamic therapy (PDT) is a therapeutic modality that can be used to ablate tumors using the localized generation of reactive oxygen species by combining a photosensitizer, light, and molecular oxygen. This modality holds promise as an adjunctive therapy in the management of colorectal cancer and could be incorporated into neoadjuvant treatment plans under the auspices of prospective clinical trials.

**Methods:**

We conducted a search of primary literature published until January 2021, based on PRISMA guidelines. Primary clinical studies of PDT for the management of colorectal cancer were included. Screening, inclusion, quality assessment, and data collection were performed in duplicate. Analyses were descriptive or thematic.

**Results:**

Nineteen studies were included, most of which were case series. The total number of patients reported to have received PDT for colorectal cancer was 137, almost all of whom received PDT with palliative intent. The most common photosensitizer was hematoporphyin derivative or Photofrin. The light dose used varied from 32 J/cm^2^ to 500 J/cm^2^. Complete tumor response (cure) was reported in 40%, with partial response reported in 43.2%. Symptomatic improvement was reported in 51.9% of patients. In total, 32 complications were reported, the most common of which was a skin photosensitivity reaction.

**Conclusions:**

PDT for the management of colorectal cancer has not been well studied, despite promising results in early clinical case series. New, well designed, prospective clinical trials are required to establish and define the role of PDT in the management of colorectal cancer.

## Background

Photodynamic therapy (PDT) is a therapeutic modality that destroys target cells using the generation of reactive oxygen species through the excitation of a photosensitizer. Photosensitizers can be administered topically or intravenously and subsequently excited by irradiation with a specific wavelength of light, typically using a laser. PDT is most commonly investigated for its ablative potential in the context of cancer and has been applied clinically to a large number of cancers, including non-melanoma skin cancer, various gastrointestinal cancers, non-small-cell lung cancer, brain cancer, breast cancer, head and neck cancer, genitourinary cancer, and more.^1^ It is particularly attractive because the mechanism by which PDT ablates tumors spares connective tissues, affecting only living cells and resulting in less scarring and anatomic distortion compared with other surgical and ablative modalities.^2^ PDT offers the opportunity to tightly target malignant tissues through a combination of localization of the photosensitizer and the directed delivery of light. Owing to the need to deliver light precisely, PDT is perhaps most readily deployed to easily accessible tumor sites, like the skin, lung, and gastrointestinal tract. A large quantity of pre-clinical data suggests that PDT can be used to ablate colorectal cancers; however, clinical translation of this data has been limited, and no photosensitizers are expressly approved, recommended, or used to treat colorectal cancer.^3^ This gap may be due to confusion surrounding the ideal treatment patient population and treatment regimen as a result of the myriad of potential variables involved. We sought to synthesize the existing clinical data in a systematic fashion, particularly with a view to clarify which patients are most likely to benefit, and what regimen is most likely to succeed. This is the first systematic review of the clinical literature investigating the use of PDT for the management of colorectal cancer.

## Methods

### Review Protocol

Our review protocol was developed a priori and registered in the international prospective register of systematic reviews (PROSPERO, CRD42021233971) on February 28, 2021.

#### Search Strategy

We conducted a systematic literature search of MEDLINE (1946–present), Medline In-Process/ePubs (daily), Embase (1947–present), Cochrane Central Register of Controlled Trials (1991–present), Cochrane Database of Systematic Reviews (2005–present), and PsycINFO (1806–present). The Web of Science (Clarivate) database was searched (1900–present). Lastly, the Scopus (Elsevier, 1960–present) database was searched. All databases were searched on the same day, Monday January 4, 2021. An update of the search was conducted on May 1, 2021, which found no new eligible studies.

The searching process followed the Cochrane Handbook^4^ and the Cochrane Methodological Expectations of Cochrane Intervention Reviews (MECIR)^5^ for conducting the search, the PRISMA guideline^6^ for reporting the search, and the PRESS guideline for peer-reviewing the search strategies^7^ drawing on the PRESS 2015 Guideline Evidence-Based Checklist used to avoid potential search errors.

Preliminary searches were conducted, and full text literature was mined for potential keywords and appropriate controlled vocabulary terms (such as Medical Subject Headings for Medline and EMTREE descriptors for Embase). The search strategy concept blocks were built on the topics of: Photodynamic Therapy AND Colorectal Cancer AND Studies. Results were limited to English language, and human subjects.

### Study Selection, Data Extraction, and Quality Assessment

Two trained reviewers (KG and LD) independently identified articles eligible for further review by performing an initial screen of identified abstracts. Articles were considered for inclusion if they reported results of human patients undergoing photodynamic therapy (i.e., administration of both a photosensitizer and a light dose) for the management of a primary colorectal cancer. Disagreement between reviewers was resolved in discussion between the two initial reviewers and a third trained reviewer (HY). Reviewers independently evaluated the quality of the studies and extracted the data. Quality assessment was performed using Joanna-Briggs Institute critical appraisal tools for use in systematic reviews, as appropriate for the study design.^8,9^

### Summarization of Data

Due to generally poor study quality and a large degree of heterogeneity in the design, reported parameters, and reported outcomes of the study, no formal statistical analysis was conducted. Descriptive numerical analyses through frequency analysis were performed where appropriate. Thematic analyses were performed where appropriate to evaluate qualitative data.

## Results

### Literature Search and Selection Process

**Figure 1.**
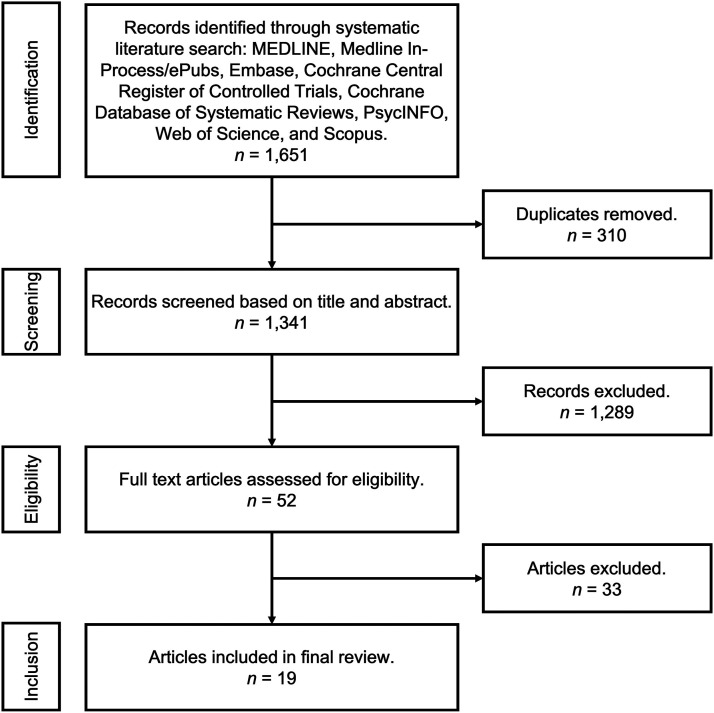
PRISMA flow diagram of citation inclusion.

Our initial search resulted in 1651 citations. After the removal of duplicate citations (310), 1341 citations were screened for relevance, of which 1289 were excluded. Of the remaining 52 studies that underwent full-text assessment for eligibility, 19 were ultimately included in the study^10-28^ (see Figure 1).

### Study Characteristics

Study characteristics are included in Table 1. Across the 19 included articles, 137 patients received PDT for colorectal cancer. Almost all studies exclusively enrolled patients for palliative indications, with tumors that were deemed “inoperable”, or who had received one or more forms of therapy in the past. The definition of “inoperable” varied slightly among studies, but typically included patients who could not receive standard of care therapies due to medical comorbidity, for anatomic reasons, or who refused the conventional therapies offered. We included 12 case series, four cohort studies, and three case reports; all were single-center studies. The median year of publication was 1995 (range 1986–2019). Most used populations with a heterogenous group of diseases, only a subset of which were patients with colorectal cancer (e.g., any gastrointestinal cancer). As a result, demographic information was not reliably available for the cohorts of patients in these studies with colorectal cancer. Four studies examined the effect of both PDT and another concurrently administered therapeutic modality (operative exploration/resection in three, polypectomy in one); all other studies examined the effect of PDT alone.

**Table 1.** Demographic and study details. PDT = photodynamic therapy.

Study	n*	Female Sex, *n* (%)	Age (Range)	Study Design	Treatment Indication	Concurrent Treatment
Herrera-Ornelas et al. 1986	11	5 (45.5)	56^b^ (40–67)	Case series	Recurrent rectal cancer	Operative resection of recurrent cancer in 5/11 patients, operative exploration in all
Jin et al. 1989	10	–	–	Case series	Advanced inoperable or recurrent gastrointestinal cancer	None
Barr et al. 1990	10	5 (50)	73^a^ (44–90)	Case series	Inoperable colorectal cancer	None
Patrice et al. 1990 (Digestive Diseases and Sciences)	16	3 (18.8)	74.5^a^ (63–88)	Case series	Inoperable gastrointestinal cancer	None
Patrice et al. 1990 (Journal of Photochemistry and Photobiology)	21	4 (19.1)	75^a,c^	Case series	Inoperable gastrointestinal cancer, lesions <40 mm in largest diameter, stage M0 only	None
Karanov et al. 1991	3	3 (100)	70^b^ (36–72)	Case series	Persistent/recurrent rectal cancer, stage T1N0M0 only, with contra-indications to other therapy	None
Kashtan et al. 1991	6	3 (50)	69^b^ (37–91)	Case series	Palliative treatment of locally advanced rectal cancer	None
Foultier et al. 1994	5	1 (20)	–	Case series	Inoperable gastrointestinal cancer	None
Allardice et al. 1994	13	5 (38.5)	63 (54–75)	Case series	Preoperative diagnosis of intra-abdominal malignancy, excluding patients with advanced malignancy	Operative resection of primary tumors as usual
Harlow et al. 1995	7	4 (57.1)	71^b^ (49–73)	Case series	Recurrent rectal adenocarcinoma following surgical ± adjuvant therapy	Operative resection of recurrent cancer
Mlkvy et al. 1995 (Neoplasma)	3	–	–	Case series	Inoperable gastrointestinal tumors	None
Mlkvy et al. 1995 (European Journal of Cancer)	1	1 (100)	45^c^	Case series	Inoperable duodenal or colorectal tumors secondary to familial adenomatous polyposis	None
Regula et al. 1995	2	–	–	Cohort study	Inoperable gastrointestinal tumors	None
Fromm et al. 1996	1	0 (0)	60^c^	Case report	Anastomotic recurrence of rectosigmoid cancer	None
Mlkvy et al. 1998	1	1 (100)	45^c^	Cohort study	Inoperable gastrointestinal tumors	None
Privalov et al. 2002	1	–	–	Cohort study	Any malignancy, standard of care therapy contraindicated	None
Nakamura et al. 2003	2	1 (2)	72^a,b,c^	Case report	Rectal cancer, recurrent or refused surgery	Snare polypectomy
Sun et al. 2016	53; 23 PDT, 30 standard care	16 (30.2); 7 (30.4) PDT, 9 (30) standard care	41.9 (23–58) PDT, 41.9 (27–56) standard care	Cohort study	Recurrent colorectal cancer	None
Zhang et al. 2019	1	0 (0)	56^c^	Case report	Rectal adenocarcinoma (T2N0M0) with positive margin on post-operative pathology, patient refused surgery	None

^a^Patients treated for colorectal cancer only.

^b^Mean.

^c^Median.

^d^Additional data unavailable.

Study quality was generally poor, with an enormous degree of heterogeneity in the design, conduct, and reporting of key methodological characteristics (Supplementary Table S1). Due to the relative dearth of evidence and similar study quality, no studies were excluded for reasons of poor quality.

### Treatment Specifications

The precise treatment parameters used in these studies varied by photosensitizer, photosensitizer dose, drug-light interval, laser excitation wavelength, light dose, and mode of light delivery (Table 2). The most common photosensitizer used was Hematoporphyrin Derivative (HpD, or similar, used in nine studies), followed by Photofrin (seven studies; note that HpD and Photofrin are essentially the same drug, but were reported differently in the primary sources, and so are being reported as such here); 5-ALA was used in three studies, and Radachlorin was used in one. HpD was typically used in doses between 2.5 mg/kg and 5 mg/kg and administered via a slow intravenous infusion. Photofrin was universally used at 2 mg/kg and administered via a comparatively more rapid IV infusion. 5-ALA was used at 30 mg/kg or 60 mg/kg and was administered orally in split doses over several hours. The drug-light interval varied based upon the photosensitizer used: HpD-PDT had a drug-light interval of 48-72 hours, Photofrin had a drug-light interval of 24-48 hours, and 5-ALA had a drug-light interval of 6 hours from the time of administration of the first dose (of the split doses).

**Table 2.** Summary of treatment parameters and outcomes by study. PDT = photodynamic therapy; HpD = hematoporphyrin derivative; ALA = aminolevulinic acid; IV = intravenous; LGIB = lower gastrointestinal bleed.

Study	Treatment Plan	Photosensitizer Treatment	Drug-Light Interval	Laser Wavelength	Method of Laser Delivery	Laser Dose	Complications	Symptom Improvement	Complete Response	Partial Response	No Response	Subjective Response	Median Survival
Herrera-Ornelas et al. 1986	Surgical exploration and resection of recurrent tumor in 5/11, followed by light delivery (intra-operative or post-operative)	HpD, 3 mg/kg IV; 2 mg/kg Photofrin	2–4 days	630 nm	External beam or interstitial irradiation, transabdominal (intra-operative) or transperineal (post-operative) fiber delivery	100–400 J/cm^2^, 100–200 mW/cm^2^, 600–3,600s per 10 cm^2^ site (external beam); 400–700 J/cm, 1000 mW, 900–2,400s per 1 cm site (interstitial irradiation)	2/11 – photosensitivity reaction	5/11	3/11	0/11	8/11	Moderate to severe inflammatory reaction with severe hemorrhagic necrosis on histology; treatment well tolerated	11 months
Jin et al. 1989	Light delivered to one or more sites based on tumor size	HpD, 5 mg/kg, IV	48–72 hours	630 nm	External beam (1–2 cm from tumor) or interstitial irradiation, transanal fiber delivery	100–250 J/cm^2^, 100–300 mW	None	Not reported	1/10	7/10	2/10	Tumor necrosis to ∼10 mm depth; degeneration and necrosis of tumor cells on histology	Not reported
Barr et al. 1990	Light delivered to up to 4 sites	HpD, 2.5 mg/kg, IV over 30 mins	48 hours	630 nm	Interstitial irradiation, transanal fiber delivery	50 J, 50–100 mW, 500-1000s	3/10 – LGIB requiring transfusion (2/10), photosensitivity reaction (1/10)	7/10	2/10	8/10	0/10	Best results with small tumors	8 months^a^
Patrice et al. 1990 (Digestive Diseases and Sciences)	Treatment/8 mm lesion site	HpD, 2.5 mg/kg or 5 mg/kg, IV	72 hours	632 nm	External beam or interstitial irradiation, transanal fiber delivery	150 J/cm^2^ or 220 J/cm^2^, 300–400 mW, 300s per 8 mm site	1/16 – stenosis requiring dilation	Not reported	8/16	5/16	3/16	Tolerance of treatment similar to standard colonoscopy	Not reported
Patrice et al. 1990 (Journal of Photochemistry and Photobiology)	1 or 2 treatments	HpD, 2.5 mg/kg or 5 mg/kg, IV	72 hours	632 nm	External beam or interstitial irradiation, transanal fiber delivery	150 J/cm^2^ or 220 J/cm^2^, 300–400 mW, 300s per 8 mm site; tip of fiber maintained between 2–2.5 cm from surface	1/21 – photosensitivity reaction, 1/21 – pain, 1/21 – edema of hepatic origin, 1/21 – bowel perforation, 1/21 – stenosis	Not reported	10/21	11/21		Tolerance of treatment similar to standard colonoscopy	25.6 months^b^
Karanov et al. 1991	2–3 irradiation sites, 1–4 sessions depending on tumor size	Hp/5, 5.1–6 mg/kg, slow IV infusion	72 hours	630 nm	External beam, transanal fiber delivery	320–400 J/cm^2^, 150–650 mW	1/3 - metrorrhagia	Not reported	3/3	0/3	0/3	White necrosis and ulceration at the treatment site; epithelialization by 10–15 days post-treatment; pale poorly vascularized mucosa at 6 months post-treatment	9 months
Kashtan et al. 1991	2 staged treatments	Photofrin, 2 mg/kg, IV infusion over 5–10 mins	24–48 hours	630 nm	External beam, transanal fiber delivery	50, 100, 150, or 200 J/cm^2^, 1000 mW, 480–2,880s	1/6 – photosensitivity reaction	1/6	2/6	3/6	1/6	Inflammatory response, friability, and edema at the treatment site	Not reported
Foultier et al. *1*994	Treated in 8 mm lesion segments	HpD, 5 mg/kg, IV over 60 mins	72 hours	632 nm	External beam (2–2.5 cm from tumor, 1 cm diameter beam), transanal fiber delivery	220 J/cm^2^, 400 mW, 300s per 8 mm site	1/5 – asymptomatic stenosis, 1/5 – mild tanning	Not reported	Not reported	Not reported	Not reported	White necrosis and ulceration at the treatment site	Not reported
Allardice et al. 1994	Irradiation of surgical bed and/or residual tumor, following surgery	HpD, 3 mg/kg, 5 mg/kg, or 111 mg/m^2^, IV	48 hours	510 nm or 630 nm	External beam, transabdominal or transperineal fiber delivery	32–63 J/cm^2^, <1,800s	2/13 – anastomotic leak, 1/13 – anastomotic leak and fistula, 1/13 – subphrenic abscess; authors state PDT unlikely to account for any complications	Not reported	8/9^c^	1/9^c^		Not reported	27 months
Harlow et al. 1995	Surgical exploration and resection of recurrent tumor, followed by light delivery (intra-operative or post-operative)	Photofrin, 2 mg/kg, IV	24–48 hours	630 nm	External beam or interstitial irradiation, transabdominal (intra-operative) or transperineal (post-operative) fiber delivery	50 J/cm^2^, 300–400 mW/cm^2^ (external beam); 300 J/cm, 300–400 mW/cm, through up to 4 fibers (interstitial irradiation)	1/7 – photosensitivity reaction, bilateral ureteric leak	Not reported	Not reported	Not reported	Not reported	Not reported	22.5 months
Mlkvy et al. 1995 (neoplasma)	2–7 interstitial spots	5-ALA, 60 mg/kg, PO	6 hours	628 nm	External beam or interstitial irradiation, transanal fiber delivery	100 J/cm^2^, 570 mW/cm of fiber (external beam); 50 J, 50 mW, or 100 J, 100 mW, 1000s per site (interstitial irradiation)	Photosensitivity reaction, nausea and vomiting, transient elevations in AST reported in overall cohort; not subdivided by tumor site	Not reported	7/7		0/7	Whitish necrosis and fibrinous exudate at treatment site; tumor necrosis to .5–1.8 mm depth	Not reported
Mlkvy et al. 1995 (European Journal of Cancer)	2–4 sites	Photofrin, 2 mg/kg, IV	48 hours	628 nm	Interstitial irradiation, transanal fiber delivery	50 J, 50 mW, or 100 J, 100 mW, 1000s per site	1/1 - photosensitivity reaction	Not reported	1/1	0/1	0/1	Whitish necrosis and fibrinous exudate at treatment site; superficial ulceration at treatment site at 1-week post-treatment, completely healed by 6 weeks post-treatment	Not reported
Regula et al. 1995	Treatment with two doses; colorectal patients received same dose, 2–7 sites	5-ALA, 30 or 60 mg/kg, PO	6 hours	628 nm or 630 nm	Interstitial irradiation, transanal fiber delivery	50 J, 50 mW, 1000s per site (interstitial irradiation, 2 sites)	Not reported	Not reported	2/2	0/2	0/2	Whitish necrosis and fibrinous exudate at treatment site	Not reported
Fromm et al. 1996	2 treatments, in one session, 13 mins between	5-ALA, 60 mg/kg, PO	6 hours	633 nm	External beam, transanal fiber delivery	50 J/cm^2^, 200 mW/cm^2^	None	Not reported	1/1	0/1	0/1	Whitish necrosis at treatment site at 9-days post-treatment; recurrence at 6 months, successfully treated with a single treatment of 100 J/cm^2^	Not reported
Mlkvy et al 1998	2–4 sites	Photofrin, 2 mg/kg, IV	48 hours	628 nm	External beam or interstitial irradiation, transanal fiber delivery	100 J/cm^2^, 570 mW/cm of fiber (external beam); 50 J, 50 mW, or 100 J, 100 mW, 1000s, per site (interstitial irradiation)	1/1 – photosensitivity reaction	Not reported	1/1	0/1	0/1	Whitish necrosis at treatment site, and sloughing of tumor; tumor down-staged to microadenoma	Not reported
Privalov et al. 2002	Single treatment	Radachlorin, .8–1.2 mg/kg, IV	1–2 hours	662 nm	External beam or interstitial irradiation, transanal fiber delivery	100–500 J/cm^2^	None	Not reported	0/1	1/1	0/1	Dense, black scab at treatment site at 1-week post-treatment; complete healing by 6-8 weeks post-treatment	Not reported
Nakamura et al. 2003	Initial polypectomy for debulking, followed by PDT 1 week later	HpD, 2.5 mg/kg, IV	48–72 hours	627.8 nm	External beam, transanal fiber delivery	150–280 mW	None	2/2	2/2	0/2	0/2	Healing ulcer seen 7-10 days after PDT, completely healed at 3 months	48.5 months
Sun et al. 2016	1–3 treatment sites PDT, no chemo-radiotherapy	Photofrin, 2 mg/kg	48 hours	630 nm	External beam, transanal fiber delivery	200 J/cm^2^, 278 mW/cm, 720s per segment, 5 mm overlap between treatment sites	6/23^d^ – fistula (2/23), photosensitivity reaction (1/23), LGIB (3/23)	12/23^e^ (52.2%)	2/23^f^	14/23^f^	7/23^f^	Length of stay decreased by 6.25 days in PDT group (*p*=.036)	6.23±1.65 months^g^
	Chemo-radiotherapy only	Adjuvant chemo-radiotherapy	Not applicable				15/30^d^ – fistula (5/30), photosensitivity (3/30), LGIB (4/30), systemic toxicity (2/30), other (1/30)	8/30^e^ (26.7%) p<.05	2/30^f^	10/30^f^	18/30^f^		3.01±1.12 months^g^
Zhang et al. 2019	2 sites	Photofrin, 2 mg/kg, IV	48 hours	630 nm	External beam, transanal fiber delivery	60 J/cm^2^ 100 mW/cm^2^, 600s per site	1/1 – stenosis requiring dilation	Not reported	1/1	0/1	0/1	Not reported	60 months

^a^1 patient missing.

^b^Median survival reported only for the 10 patients who experienced a complete response.

^c^Outcomes reported for only 9 patients with colorectal cancer.

^d^*p* = 0.031.

^e^*p* < 0.05.

^f^*p* = .035.

^g^*p* = .013.

**Figure 2.**
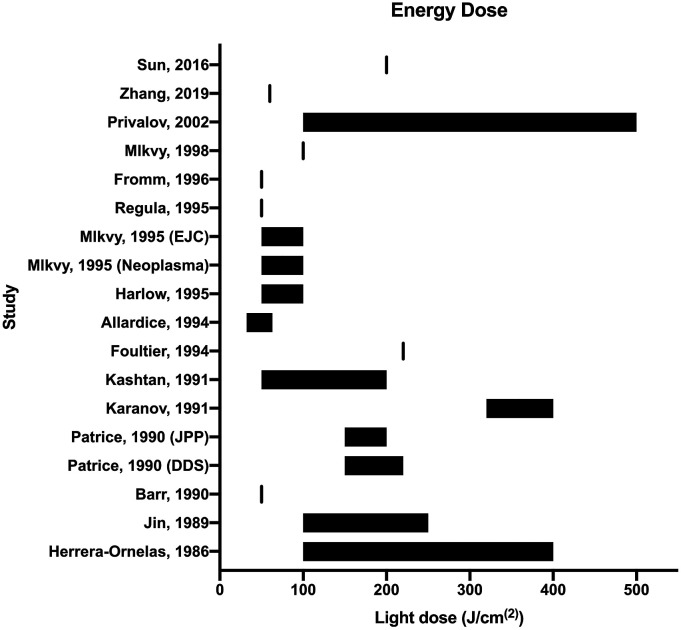
Forest plot of light energy doses used in the included studies.

All studies except for two used a laser excitation wavelength around 630 nm (Allardice et al.^19^ used 510 nm or 630 nm for HpD, and Privalov et al.^12^ used 662 nm for their Radachlorin photosensitizer). Light was administered using one of two methods: either external beam irradiation (in which a beam of laser light is directed onto the tumor using a fiber optic) or interstitial irradiation (in which a fiber optic with a cylindrical diffuser is introduced into the tumor parenchyma). Overall, more studies used external beam irradiation compared with interstitial irradiation (15 vs 11); however, eight studies used a combination of both, with eight performing external beam irradiation only, and three performing interstitial irradiation only. All but three studies delivered the fiber optic transanally via an endoscope; the remaining studies administered light concurrently with transabdominal surgery and either delivered light intraoperatively, placed fiber optics during surgery that were later used to deliver light, or introduced fiber optics via the perineal wound following an abdominoperineal resection. The light dose delivered varied between studies from 32 J/cm^(2)^–500 J/cm^(2)^, with the most common light dose falling around at ∼200 J/cm^(2)^ (see Figure 2). The power varied from 50 mW/cm^(2)^–1000 mW/cm^(2)^. Treatment time varied from 300s (5 mins) to 3,600s (60 mins).

### Outcomes

Complications of treatment were reported in 18 studies, with four reporting no complications at all. The most commonly reported complication was skin photosensitivity (usually manifested as a superficial burn upon exposure to sunlight, reported in at least nine patients); other common complications included lower gastrointestinal bleed (five patients), and stenosis (variably requiring dilation, four patients). In addition, five patients were reported to have suffered a fistula of some kind, one patient was reported to have suffered a bowel perforation, and another was reported to have suffered from a ureteric leak requiring ureteral stenting; however, this was in a patient who had received an abdominoperineal resection and received trans-perineal light delivery. In total, 32 complications were reported. The only study to compare a cohort of patients treated with PDT to a cohort treated without, was Sun et al.^10^ who reported a complication rate of 26.1% in PDT treated patients compared with 50% in patients undergoing standard of care adjuvant chemo-radiotherapy (*p* = .031).

Five studies reported on symptomatic improvement, with all studies reporting at least some degree of symptom improvement (specific symptoms varied, but included bleeding, obstruction, and pain) in patients who underwent PDT for colorectal cancer. In aggregate, 27/52 (51.9%) reported patients experienced symptomatic improvement. The only study to compare a cohort of patients treated with PDT to a cohort treated without, was Sun et al.*,*^10^ who found that 52.2% of patients treated with PDT experienced symptom improvement compared with 26.7% of patients treated with standard of care adjuvant chemo-radiotherapy (*p* < .05).

### Tumor Response

Studies variably reported tumor response to PDT; two studies did not report any tumor response outcomes (Table 2). In those reporting tumor response, response was reported as complete, partial, or no response (though not all studies reported all of these categories). Among those reporting complete response (defined as complete regression of the tumor at any point during follow-up), the complete response rate was 46/115 (40%). Among those reporting partial response (variably defined as incomplete regression or temporary growth arrest of the tumor), the partial response rate was 38/88 (43.2%). Complete or partial response was reported in 82/103 (79.6%) of patients with reporting of such responses. No response was reported in 21/95 (22.1%) of patients with reporting of no response. The only study to compare a cohort of patients treated with PDT to a cohort treated without, was Sun et al.*,*^10^ who found a greater rate of complete and partial response in the PDT treated group as compared to the standard of care group (8.7% vs 6.7% and 60.9% vs 33.3%, respectively).

Sixteen studies reported subjective responses to PDT treatment, with eight reporting white necrosis, four reporting superficial ulceration, and three reporting fibrinous exudate at the treatment site within the first week of treatment. Necrosis was commonly seen on histological assessment of any lesion biopsies. Complete healing was reported as early as 6 weeks post-treatment in two studies. In addition, three studies reported that tolerance of treatment was comparable to that of endoscopy. Two studies reported on the effective treatment depth, with one reporting a range between 5 and 18 mm in depth of necrosis,^17^ and the second reporting necrosis to ∼10 mm^26^; similarly, Barr et al.^25^ reported that subjectively, smaller tumors were more likely to be ablated.

Median survival was reported in nine studies, with a median of 22.5 months (range 6.23–60). Sun et al.^10^ directly compared a cohort receiving PDT to another not receiving PDT, finding a greater median survival in PDT treated patients compared to those receiving standard of care therapy (6.23 ± 1.65 months vs 3.01 ± 1.12 months, *p* = .013).

## Discussion

Photodynamic therapy is a relatively novel treatment modality that has been thoroughly demonstrated in both pre-clinical and clinical studies to be capable of tumor ablation, yet it remains poorly utilized in clinical practice despite its promise for many modern and growing applications, including in the management of rectal cancer. Currently, almost all clinical PDT for cancer is conducted using Photofrin (Pinnacle Biologics) as a photosensitizer and using a laser assembly distributed by the same company for the management of certain endobronchial and esophageal tumors. Another significant clinical application of PDT is in the management of non-melanoma skin cancer, where the photosensitizer is applied topically.

Reasons for the generally poor uptake of PDT as a modality are frequently discussed in the PDT literature but can be summarized generally as challenges related to the complexity of the therapy. PDT relies upon delivery of the correct dose of a photosensitizing agent (usually administered intravenously) to a tumor, followed by irradiation at a specific time-point following drug administration, with a particular wavelength and power output light, for a specific period of time, via either external beam irradiation or interstitial irradiation, at one or more sites. This entire procedure may then be repeated any number of times. Even if all of these parameters can be achieved and consistently delivered to patients, the therapeutic effect may not be consistent between patients due to variation in the size and shape of both the tumor and the patient, as well as differences in tissue pigmentation.

All of these complexities in treatment plan are reflected in the vast heterogeneity of the treatment parameters used in the studies analyzed in this article. The various attempts of the authors to modify their protocol—either *ad hoc* or *post hoc*—can be seen in Table 2. For instance, Barr et al.*,*^25^ Mlkvy et al.*,*^13,16,17^ and Patrice et al.^23,24^ appear to have changed their light dose parameters mid-way through the study, and Kashtan et al.^21^ designed a somewhat complex “step-up” protocol to increase their light dose depending upon the observed effect. All of this reflects the complexity involved in optimizing PDT for the management of colorectal cancer.

We found only one reasonably well-conducted study that makes a meaningful comparison between PDT and a control group^10^; the remaining studies were extremely heterogenous in terms of study population, treatment parameters, and measured outcomes. In addition, they were generally smaller studies with limited statistical power. Despite these drawbacks, these studies provide compelling reasons to believe that PDT is a viable therapeutic modality that can be deployed to great effect in patients with colorectal cancer. We found that 79.6% of patients in these studies experienced at least a partial tumor response to therapy, with 40% experiencing a complete ablation of the tumor. In addition, 51.9% of patients reported symptom improvement following PDT, with a reasonable safety profile. All of these results must be understood while bearing in mind that all of these trials were conducted on patients who had no other viable treatment options, thereby underestimating the true therapeutic potential of PDT. These promising early results call for a more methodologically and statistically robust clinical study of PDT in a dedicated and well-defined colorectal cancer patient population.

Future clinical studies of colorectal PDT must look to previous work for guidance when determining the most scientifically robust methodology, and despite the heterogeneity seen in these studies, some common themes emerge. Firstly, the most commonly used photosensitizer used was HpD or Photofrin (largely identical), with Photofrin being the most readily available agent on the market. Secondly, a dose of 2 mg/kg, a laser wavelength of ∼630 nm, and a drug-light interval of 24–48 hours was universally used for Photofrin PDT. Light delivery is the most challenging and variable component of PDT; however, Photofrin trials typically deliver a light dose between 50 and 100 J/cm^2^ with a power between 100 and 500 mW/cm^2^. The optimal method of light delivery remains uncertain, with many studies employing both external beam and interstitial irradiation; this reflects the ongoing conflict between the perhaps more scientifically robust interstitial irradiation method and the more pragmatic external beam irradiation approach. The decision between these methods must be made based on the expertise and comfort of the local clinicians and medical biophysicists. These parameters can form the basis for the methodology of future studies seeking to perform PDT, particularly for colorectal cancer.

Clinicians’ interest in PDT for colorectal cancer was at a height two decades ago and has since waned, with a corresponding rapid advance in other non-surgical treatment options like chemotherapy and radiotherapy. However, given the recent interest in total neoadjuvant and sphincter-preserving therapy, it is no longer possible for oncologists to ignore the potential therapeutic benefits offered by PDT in good conscience. PDT has the potential to be used in combination with other neoadjuvant, adjuvant, and non-operative therapies to manage colorectal cancer. Further large-scale, prospective, randomized, clinical trials are required before PDT can be fully integrated into the treatment pathway for colorectal cancer; however, the ability to repeat PDT indefinitely and ablate tumors in an extremely precise and targeted fashion with limited off-target toxicity makes it an extremely attractive tool to add to the oncologist’s arsenal. We hope that this review can generate interest in PDT as an adjunctive ablative modality for the management of colorectal cancer and can help to guide future clinicians and researchers in the conduct of better-designed studies.

## Supplemental Material

sj-pdf-1-sri-10.1177_15533506221083545 – Supplemental Material for Photodynamic Therapy for Colorectal Cancer: A Systematic Review of Clinical Research Click here for additional data file. Supplemental Material, sj-pdf-1-sri-10.1177_15533506221083545 for Photodynamic Therapy for Colorectal Cancer: A Systematic Review of Clinical Research by Keegan Guidolin, Lili Ding, Han Yan, Marina Englesakis HBA, Sami Chadi, Fayez Quereshy and Gang Zheng in Surgical Innovation

## Appendix 1

### Medline Search Strategy Ovid MEDLINE(R) 1946 to December 31, 2020.

**Table:**

#	Searches
1	exp Colorectal Neoplasms/
2	(adenocarcinom* adj3 (colorect* or colon* or rect* or intestine* or large bowel* or bowel* or anal or anus or perianal or peri-anal or circumanal or sigmoid*)).mp,kw.
3	(adenom* adj3 (colorect* or colon* or rect* or intestine* or large bowel* or bowel* or anal or anus or perianal or peri-anal or circumanal or sigmoid*)).mp,kw.
4	(cancer* adj3 (colorect* or colon* or rect* or intestine* or large bowel* or bowel* or anal or anus or perianal or peri-anal or circumanal or sigmoid*)).mp,kw.
5	(carcinom* adj3 (colorect* or colon* or rect* or intestine* or large bowel* or bowel* or anal or anus or perianal or peri-anal or circumanal or sigmoid*)).mp,kw.
6	(malignan* adj3 (colorect* or colon* or rect* or intestine* or large bowel* or bowel* or anal or anus or perianal or peri-anal or circumanal or sigmoid*)).mp,kw.
7	(metasta* adj3 (colorect* or colon* or rect* or intestine* or large bowel* or bowel* or anal or anus or perianal or peri-anal or circumanal or sigmoid*)).mp,kw.
8	(neoplas* adj3 (colorect* or colon* or rect* or intestine* or large bowel* or bowel* or anal or anus or perianal or peri-anal or circumanal or sigmoid*)).mp,kw.
9	(tumor* adj3 (colorect* or colon* or rect* or intestine* or large bowel* or bowel* or anal or anus or perianal or peri-anal or circumanal or sigmoid*)).mp,kw.
10	(tumour* adj3 (colorect* or colon* or rect* or intestine* or large bowel* or bowel* or anal or anus or perianal or peri-anal or circumanal or sigmoid*)).mp,kw.
11	or/1-10 [ Colon / Rectal / Colorectal Cancer ]
12	exp Photochemotherapy/
13	photosensitizing agents/ or 5-methoxypsoralen/ or aminolevulinic acid/ or dihematoporphyrin ether/ or ficusin/ or furocoumarins/ or hematoporphyrin derivative/ or hematoporphyrins/ or methoxsalen/ or trioxsalen/ or verteporfin/
14	Phototherapy/
15	exp Hematoporphyrins/
16	photodynamic therap*.mp.
17	photo-dynamic therap*.mp.
18	photochemotherap*.mp.
19	photo-chemotherap*.mp.
20	photoradiat*.mp.
21	photo-radiat*.mp.
22	photosensitiz*.mp.
23	photosensitis*.mp.
24	phototherap*.mp.
25	photo-therap*.mp.
26	nanophotosensiti*.mp.
27	nano-photosensiti*.mp.
28	XPDT.mp.
29	"X-PDT".mp.
30	photoactivat*.mp.
31	photo-activat*.mp.
32	129497-78-5.rn.
33	133513-12-9.rn.
34	136752-88-0.rn.
35	14459-29-1.rn.
36	274679-00-4.rn.
37	87806-31-3.rn.
38	"CL 184,116".mp.
39	"CL 184116".mp.
40	"CL 318,952".mp.
41	Haemaporphyrin??.mp.
42	Haematoporphyrin??.mp.
43	Hamatoporphyrin??.mp.
44	Hematoporphyrin??.mp.
45	Hemedomine??.mp.
46	Methoxsalen??.mp.
47	U4VJ29L7BQ.rn.
48	palladium-bacteriopheophorbide?.mp.
49	Pd-bacteriopheophorbide?.mp.
50	Pd-Bpheid??.mp.
51	Photodyn??.mp.
52	"Photosan 3".mp.
53	Polyhematoporphyrin??.mp.
54	Porfimer??.mp.
55	Porfimere??.mp.
56	Porfimerum??.mp.
57	Tookad??.mp.
58	Trioxsalen??.mp.
59	Y6UY8OV51T.rn.
60	Verteporfin??.mp.
61	0X9PA28K43.rn.
62	verteporphin??.mp.
63	Visudyne??.mp.
64	"WST 09".mp.
65	"WST-09".mp.
66	"WST09".mp.
67	Y3834SIK5F.mp.
68	298-81-7.rn.
69	3902-71-4.rn. [trioxsalen]
70	8mop.mp.
71	8-mop.mp.
72	Ammoidin??.mp.
73	Bergapten??.mp.
74	bpd-ma??.mp.
75	Deltasoralen??.mp.
76	Dermox??.mp.
77	Geroxalen??.mp.
78	Meladinina??.mp.
79	Meladinine??.mp.
80	Meloxine??.mp.
81	Methoxa-dome??.mp.
82	Methoxy psoralen??.mp.
83	Methoxypsoralen??.mp.
84	nsc 71047??.mp.
85	nsc71047??.mp.
86	Oxsoralen??.mp.
87	Pentaderm??.mp.
88	Puvalen??.mp.
89	Trimethylpsoralen??.mp.
90	Trioxisalenum??.mp.
91	Trioxysalen??.mp.
92	Trisoralen??.mp.
93	Ultramop??.mp.
94	Xanthotoxin??.mp.
95	or/12-94 [ Photodynamic Therapy & related terms ]
96	11 and 95 [ CRC + Photodynamic Therapy ]
97	limit 96 to english language
98	(animal or animals or ape or apes or baboon or baboons or bat or bats or bird or birds or boar or boars or bonobo or bonobos or bovine or camel or camels or canine or canines or cat or cats or cattle or chicken or chickens or chimpanzee or chimpanzees or dog or dogs or dromedary or dromedaries or duck or ducks or equine or equines or feline or felines or ferret or ferrets or frog or frogs or fowl or fowls or goat or goats or hare or hares or hen or hens or horse or horses or lamb or lambs or livestock or macaque or macaques or mandrill or mandrills or mice or mink or minks or monkey or monkeys or mouse or murine or ovine or pig or pigs or piglet or piglets or poultry or porcine or orangutan or orangutans or rabbit or rabbits or rat or rats or rodent or rodents or sheep or swine or tamarin or tamarins or tiger or tigers or veterinary or veterinarian or veterinarians or waterfowl or waterfowls or weasel or weasels or veterinar* or (veterinar* or fish or shellfish)).ti,jw.
99	97 not 98
100	exp animals/ not (exp animals/ and exp humans/)
101	97 not 100
102	limit 97 to humans
103	99 or 101 or 102
104	remove duplicates from 103
105	Clinical Trial, Phase III/
106	exp Clinical Trial/
107	Clinical Trials, Phase III as Topic/
108	Comparative Study/
109	Controlled Clinical Trial/
110	Controlled Clinical Trials as Topic/
111	Cross-Sectional Studies/
112	Double-Blind Method/
113	Equivalence Trial/
114	Equivalence Trials as Topic/
115	exp Case-Control Studies/
116	exp Cohort Studies/
117	exp Randomized Controlled Trial/
118	exp Randomized Controlled Trials as Topic/
119	Longitudinal Studies/
120	Meta-Analysis as Topic/
121	Meta-Analysis/
122	Multicenter Studies as Topic/
123	Multicenter Study/
124	Observational Study/
125	Observational Studies as Topic/
126	Placebos/
127	Pragmatic Clinical Trial/
128	Pragmatic Clinical Trials as Topic/
129	Prospective Studies/
130	Retrospective Studies/
131	Systematic Review/
132	Systematic Reviews as Topic/
133	Validation Studies/
134	("phase 1" or "phase1" or "phase I").mp.
135	("phase 2" or "phase2" or "phase II").mp.
136	("phase 3" or "phase3" or "phase III").mp.
137	((multicenter* or multicentre*) adj2 (trial? or study or studies)).mp.
138	((noninferiority or non-inferiority) adj4 (trial? or study or studies)).mp.
139	((single or double or triple or treble) adj3 (blind* or mask*)).mp.
140	(case control* adj2 (study or studies)).mp.
141	(comparative adj2 (trial? or study or studies)).mp.
142	(conceal* adj2 allocat*).mp.
143	(controlled adj1 clinical adj2 (trial? or study or studies)).mp.
144	(cross-sectional* adj2 (study or studies)).mp.
145	(equivalen* adj4 (trial? or study or studies)).mp.
146	(evaluation adj1 (study or studies)).mp.
147	(longitudinal* adj2 (study or studies)).mp.
148	(meta-anal* or metanal* or metaanal*).mp.
149	(observational adj2 (trial? or study or studies)).mp.
150	(overview? adj4 (review or reviews)).mp.
151	(pragmatic adj2 (trial? or study or studies)).mp.
152	(prospective* adj2 (study or studies)).mp.
153	(retrospective* adj2 (study or studies)).mp.
154	(superiority adj4 (trial? or study or studies)).mp.
155	(systematic adj4 (review or reviews or overview or overviews)).mp.
156	(validation adj1 (study or studies)).mp.
157	cohort*.mp.
158	placebo*.mp.
159	quantitative*.mp.
160	quasirandom*.mp.
161	random*.mp.
162	research*.hw,pt.
163	semiquantitative.mp.
164	Feasibility Studies/
165	(feasibility adj2 (study or studies)).mp.
166	Pilot Projects/
167	(pilot adj2 (project? or study or studies or trial?)).mp.
168	or/105-167 [ Studies ]
169	104 and 168 [ CRC + Photodynamic Therapy + Studies ]
