# Supplementary material for: Photodynamic Therapy for Colorectal Cancer: A Systematic Review of Clinical Research
Source: Surg Innov. 2022 Apr 15;29(6):788–803. doi: 10.1177/15533506221083545 (PMC9667091; doi:10.1177/15533506221083545)
Supplement: sj-pdf-1-sri-10.1177_15533506221083545 – Supplemental Material for Photodynamic Therapy for Colorectal Cancer: A Systematic Review of Clinical Research [file sj-pdf-1-sri-10.1177_15533506221083545.pdf]

| Case Series | | | | | | | | | | | | |
| --- | --- | --- | --- | --- | --- | --- | --- | --- | --- | --- | --- | --- |
| Article | Were there clear inclusion criteria? | Was the condition measured in a standard, reliable way for all participants included in the case series? | Were valid methods used for identification of the condition for all participants included in the case series? | Did the case series have consecutive inclusion of participants? | Did the case series have complete inclusion of participants? | Was there clear reporting of the demographics of the participants in the study? | Was there clear reporting of the clinical information of the participants? | Were the outcomes of follow-up results of cases clearly reported? | Was there clear reporting of the presenting site(s)/clinic(s) demographic information? | Was statistical analysis appropriate? | Overall appraisal |  |
| Herrera-Ornelas *et al*, 1986 | Yes | Yes | Yes | No | Unclear | Yes | Yes | Yes | No | N/A | Include |  |
| Jin *et al*, 1989 | No | No | No | No | No | No | No | No | Yes | N/A | Include |  |
| Barr *et al*, 1990 | Unclear | Yes | Unclear | Unclear | Unclear | No | Yes | Yes | No | N/A | Include |  |
| Patrice *et al*, 1990 (Dig) | Yes | Yes | Yes | No | No | Yes | Yes | Unclear | No | N/A | Include |  |
| Patrice *et al*, 1990 (JPP) | Yes | Yes | Yes | Unclear | Yes | Yes | Yes | Yes | No | N/A | Include |  |
| Karanov *et al*, 1991 | Yes | Yes | Yes | Unclear | Yes | Yes | Unclear | Yes | No | N/A | Include |  |
| Kashtan *et al*, 1991 | Yes | Yes | Yes | Unclear | Unclear | Yes | No | Yes | No | N/A | Include |  |
| Foultier *et al*, 1994 | Yes | Yes | Yes | No | Yes | Yes | Yes | Yes | No | N/A | Include |  |
| Allardice *et al*, 1994 | Unclear | No | Unclear | Yes | Unclear | Yes | Yes | No | No | N/A | Include |  |
| Harlow *et al*, 1995 | Yes | Yes | Yes | No | No | Yes | Yes | Yes | Yes | N/A | Include |  |
| Mlkvy *et al*, 1995 (neo) | Yes | Unclear | Unclear | Unclear | Unclear | Yes | Unclear | Yes | No | N/A | Include |  |
| Mlkvy *et al*, 1995 (EJC) | Yes | Unclear | Unclear | Unclear | Unclear | Yes | Yes | Yes | No | N/A | Include |  |
| Regula *et al*, 1995 | Unclear | Unclear | No | Unclear | No | No | No | Yes | No | N/A | Include |  |
| Mlkvy *et al*, 1998 | Unclear | No | Unclear | No | No | Unclear | Unclear | Unclear | No | N/A | Include |  |
| Privalov *et al*, 2002 | No | No | No | Unclear | No | No | No | No | No | N/A | Include |  |
| Cohort Studies | | | | | | | | | | | | |
| Article | Were the two groups similar and recruited from the same population? | Were the exposures measured similarly to assign people to both exposed and unexposed groups? | Was the exposure measured in a valid and reliable way? | Were confounding factors identified? | Were strategies to deal with confounding factors stated? | Were the groups/participants free of the outcome at the start of the study (or at the moment of exposure)? | Were the outcomes measured in a valid and reliable way? | Was the follow-up time reported and sufficient to be long enough for outcomes to occur? | Was follow-up complete, and if not, were the reasons to loss to follow-up described and explored? | Were strategies to address incomplete follow-up utilized? | Was appropriate statistical analysis used? | Overall appraisal |
| Sun *et al*, 2016 | Unclear | Yes | Unclear | No | No | Yes | Yes | No | Yes | No | Yes | Include |
| Case Reports | | | | | | | | | | | | |
| Article | Were patient's demographic characteristics clearly described? | Was the patient's history clearly described and presented as a timeline? | Was the current clinical condition of the patient on presentation clearly described? | Were diagnostic tests or assessment methods and the results clearly described? | Was the intervention(s) or treatment procedure(s) clearly described? | Was the post-intervention clinical condition clearly described? | Were adverse events or unanticipated events identified and described? | Does the case report provide takeaway lessons? | Overall appraisal |  |  |  |
| Fromm *et al*, 1996 | Yes | Yes | Yes | Yes | Yes | Yes | Yes | Yes | Include |  |  |  |
| Nakamura *et al*, 2003 | Yes | No | Yes | Yes | Yes | Yes | Yes | Yes | Include |  |  |  |
| Zhang *et al*, 2019 | Yes | No | Yes | Yes | Yes | Unclear | Yes | Yes | Include |  |  |  |

Table S1. Study quality assessment according to the appropriate published JBI scoring systems.
